# When will RNA get its AlphaFold moment?

**DOI:** 10.1093/nar/gkad726

**Published:** 2023-09-13

**Authors:** Bohdan Schneider, Blake Alexander Sweeney, Alex Bateman, Jiri Cerny, Tomasz Zok, Marta Szachniuk

**Affiliations:** Institute of Biotechnology of the Czech Academy of Sciences, Prumyslova 595, CZ-252 50 Vestec, Czech Republic; European Molecular Biology Laboratory, European Bioinformatics Institute (EMBL-EBI), Wellcome Genome Campus, Hinxton, CB10 1SD, UK; European Molecular Biology Laboratory, European Bioinformatics Institute (EMBL-EBI), Wellcome Genome Campus, Hinxton, CB10 1SD, UK; Institute of Biotechnology of the Czech Academy of Sciences, Prumyslova 595, CZ-252 50 Vestec, Czech Republic; Institute of Computing Science and European Centre for Bioinformatics and Genomics, Poznan University of Technology, Piotrowo 2, 60-965 Poznan, Poland; Institute of Computing Science and European Centre for Bioinformatics and Genomics, Poznan University of Technology, Piotrowo 2, 60-965 Poznan, Poland; Institute of Bioorganic Chemistry, Polish Academy of Sciences, Noskowskiego 12/14, 61-704 Poznan, Poland

## Abstract

The protein structure prediction problem has been solved for many types of proteins by AlphaFold. Recently, there has been considerable excitement to build off the success of AlphaFold and predict the 3D structures of RNAs. RNA prediction methods use a variety of techniques, from physics-based to machine learning approaches. We believe that there are challenges preventing the successful development of deep learning-based methods like AlphaFold for RNA in the short term. Broadly speaking, the challenges are the limited number of structures and alignments making data-hungry deep learning methods unlikely to succeed. Additionally, there are several issues with the existing structure and sequence data, as they are often of insufficient quality, highly biased and missing key information. Here, we discuss these challenges in detail and suggest some steps to remedy the situation. We believe that it is possible to create an accurate RNA structure prediction method, but it will require solving several data quality and volume issues, usage of data beyond simple sequence alignments, or the development of new less data-hungry machine learning methods.

## Graphical Abstract

**Graphical Abstract:**
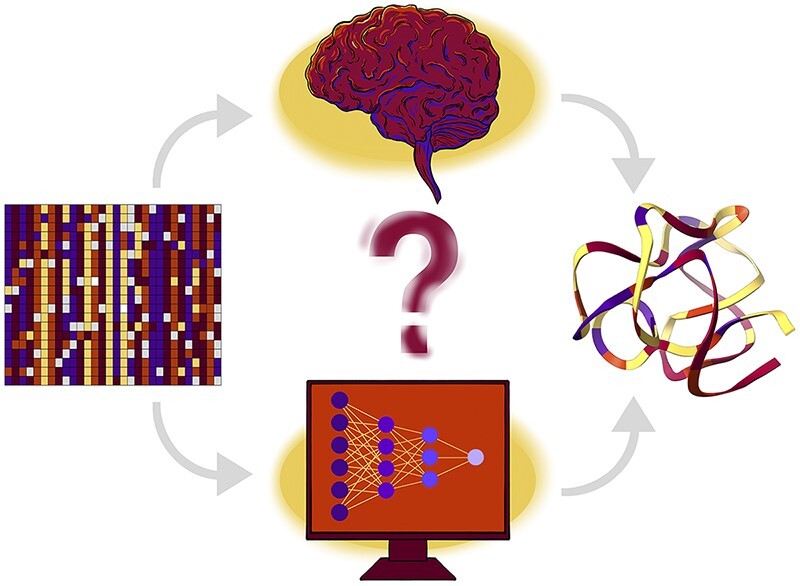

## INTRODUCTION

RNA molecules play many key functions within cells. Perhaps the most striking example is in translation, where it has been shown that the ability to build proteins is orchestrated by ribosomal particles, with the crucial catalytic step being performed by the ribosomal RNA itself, with amino acid residues delivered specifically by transfer RNAs. Untranslated regions of mRNAs and viruses harbor numerous regulatory elements. There are also a large number of noncoding RNAs (ncRNA) for which, despite decades of research, we have only a scant understanding of their functions. An example is the large class of long noncoding RNAs in animal genomes. These RNA genes are numerous, perhaps exceeding the number of protein-coding genes and seem to play a range of subtle regulatory roles (1). Many ncRNA functions depend on the stable (ribosome, tRNA) or transient (spliceosome) structure of RNA. Knowledge of RNA structures can answer basic scientific questions and can be of great help in design of new types of drugs and therapies. Structures can help answering the fundamental question of evolution whether life started with RNA as ‘RNA World’ (2) or other, perhaps peptide-type molecules. Rational drug design would without a doubt benefit from reliable predictions of RNA structures. Increasingly, the growing issue of bacterial drug resistance is approached from different perspectives but specific inhibition of ribosome particles offers a promising route to effective treatment (3). RNA therapies are attracting more attention from large pharmaceutical companies (4).

**Figure 1.**
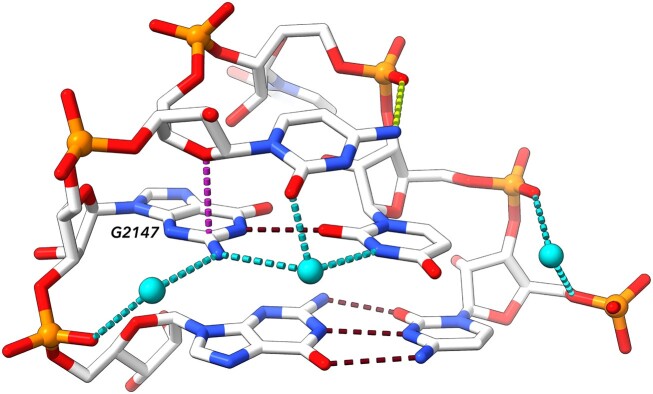
Examples of interactions in an RNA molecule. Some of the most important interactions are highlighted in dashed lines: base pairing hydrogen bonds in dark red, sugar-base stacking in dark violet, phosphate-base hydrogen bond in yellow, water-formed hydrogen bonds in cyan (waters are depicted as cyan balls). The bottom pair is canonical Watson–Crick, the pair above is a G–U pair ‘locked’ by interaction with bridging water molecule. G2147 is in *syn* orientation and dinucleotide C2146–G2147 is in the left-handed Z-form conformation (note the inverted direction of the ribose of C2146 further stabilized by stacking its O4’ to the guanine aromatic ring). Displayed is a six nucleotide loop from 80 nucleotide long fragment of 23S RNA from *Thermus thermophilus* complexed with ribosomal protein L1 (PDB ID: 4qvi) (5).

RNA building blocks, nucleotides, are chemically complex with aromatic nitrogenous bases, chiral ribose sugar rings and phosphate groups. The bases are able to stack on each other by van der Waals interactions, but they also carry large electrical moments and can form strong hydrogen bonds. Ribose rings strongly constrain backbone geometries by their puckers; the C3’-endo pucker prevails in RNA, but a ribose can also locally adopt the C2’-endo pucker, thus radically changing the backbone geometry. The phosphate groups are perhaps structurally the most complex parts of the RNA molecules due to d-orbitals in phosphorous atoms. Both torsion angles describing the conformations around the phosphodiester bonds O3’-P and P-O5’ called ζ and α prefer -gauche orientations, but the torsions can adopt any other combinations of gauche, trans and -gauche (+60°, 180° and –60°) conformations. Phosphates in nucleic acids under normal conditions are charged and render whole RNA or DNA molecules strongly negative, which needs to be neutralized by interacting positive ions. The single negative charge of each phosphate is distributed between its unbound oxygen atoms that are highly polarizable and capable of forming hydrogen bonds to other RNA atoms, proteins and water, but also of forming charge-charge interactions to amino acids, other cellular components such as amines and prominently also to metals. All intra- and inter-molecular interactions in which RNA molecules are involved determine their structures. Figure 1 illustrates at least some of these physically complex interactions as they were observed in a small six-nucleotide loop from an 80-nt fragment of rRNA from a crystal structure 4qvi (5).

## RNA 3D STRUCTURE PREDICTION: STATE OF THE ART

In the 1960s, first attempts began to reconstruct *in silico* the 3D structures of RNA molecules based on sequence homology (6). These efforts became more frequent with a growing number of experimentally determined 3D RNA structures. Building *in silico* models relied largely on manual manipulation of structure templates in a computational environment. The first interactive tool targeting RNA tertiary structure modeling was published in 1998 (7). Several years later, systems that could fully or semi-automatically process from RNA sequence to a 3D model began to appear, using *ab initio* folding such as FARFAR (8), iFoldRNA (9), NAST (10), SimRNA (11) and Vfold (12); or homology modeling such as RNABuilder (13) and ModeRNA (14), or a fragment-based assembly approach used in MC-Fold/MC-Sym (15), Assemble (16), RNAComposer (17) and 3dRNA (18). In the past two years, deep learning (DL)-based predictive models have begun to emerge. The paper by Townshend *et al.* (19) presented a DL model that predicted the quality (RMSD) of a new computer-generated 3D RNA structure. Meanwhile, other works (20–22) described methods that used deep learning for the end-to-end 3D prediction of the RNA structure.

**Figure 2.**
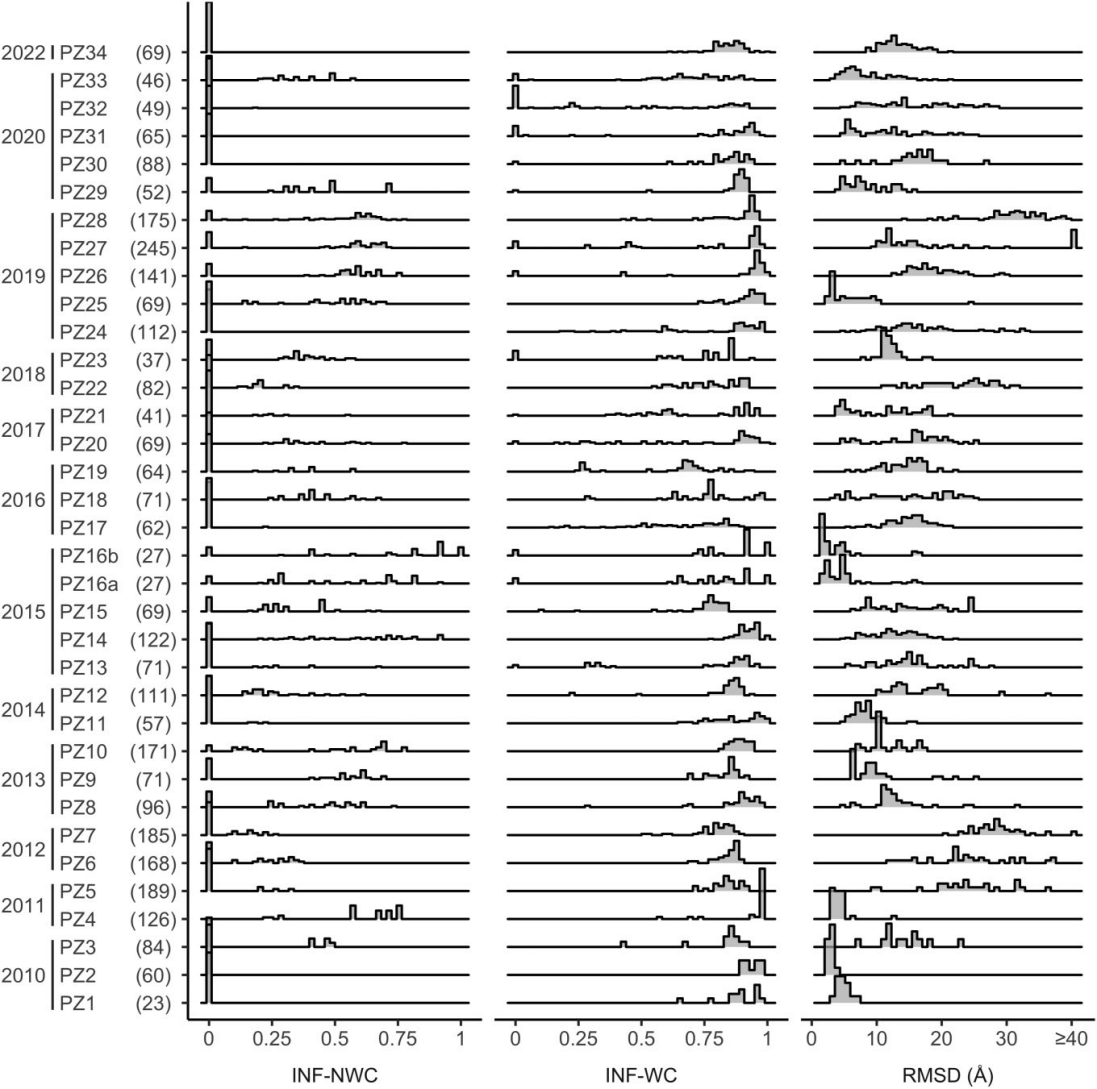
Distribution of values of selected evaluation measures for the predictions submitted to RNA-Puzzles from inception to 2022. Numbers in parentheses next to each puzzle indicate the total number of nucleotides for all structures in each puzzle.

With the increasing availability of computer-based methods for predicting 3D RNA structures, the question of the reliability and quality of the generated models became more important. In response, RNA-Puzzles, a collective blind experiment to critically evaluate the prediction of 3D RNA structures, was started in 2010 (23). During the past 12 years, RNA-Puzzles organized 38 competitive challenges (24) and two dedicated projects—modeling structures from unknown Rfam families and untranslated region of SARS-CoV-2 (25). Within each, participants predicted the tertiary structure of a single RNA target. The predictions were evaluated mainly by comparing them with a reference structure, once the latter was published in the Protein Data Bank and the assessments for 34 challenges are currently known (data as of February 2023). Several similarity and distance measures were used for evaluation, some of which were specifically developed for RNA (26–30). For example, Interaction Network Fidelity (INF), a similarity measure, scores the prediction of base pairs, Watson–Crick (INF-WC), non-Watson–Crick (INF-NWC) and stacking (INF-stacking). As shown in Figure 2, during the 12 years of challenges in RNA-Puzzles, INF-WC generally ranged between 0.75 and 1.0, demonstrating that most models had accurately predicted double helical stem motifs (INF = 1 means ideal prediction and 0 is failure). However, INF-NWC scored close to 0 for most predictions, which is of concern since non-Watson–Crick base pairs play a crucial role in determining the overall fold of the RNA, influencing stem packing and junction topologies. RMSD indicates how the predicted 3D coordinates diverge from those of the reference structure and shows only a few models with RMSD<5Å. For most RNA-Puzzles, the distribution of RMSD values is multimodal and spreads over a wide range. Therefore, despite significant advances in modeling approaches, predicting RNA coordinates with native-like features remains challenging and requires improvements in both accuracy and quality (31).

**Figure 3.**
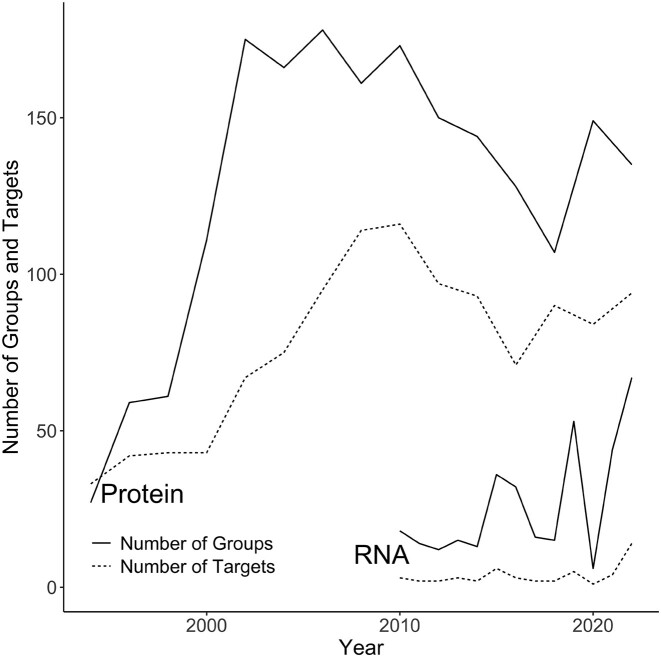
Numbers of RNA and protein structure predictions made in RNA-Puzzles and CASP competitions. The solid lines represent the numbers of groups competing in CASP and RNA-Puzzles; the dashed lines are for the number of protein/RNA targets. From 2010 to 2021, RNAs were predicted only in RNA-Puzzles and in 2022, CASP included also RNA targets, which is responsible for the recent spike in targets and groups involved in 3D RNA structure prediction.

The RNA-Puzzles initiative has adopted many mechanisms that were developed in CASP, the biennial experiment for the critical assessment of protein structure prediction. The first CASP competition was launched in 1994 (32), a quarter of a century after pioneering research into 3D computer modeling of protein structure began (33). Twenty-seven participating groups were challenged to predict the atomic coordinates of 33 amino acid sequences. In subsequent editions of CASP, the number of targets and participants increased (Figure 3), and new competition categories emerged. This included a fully automatic prediction by web servers, a category that started in 2000 (CASP4). Eighteen years later, AlphaFold (34) entered the game in CASP13 (35) to make a breakthrough in protein structure prediction in 2020 (CASP14) (36). RNA-Puzzles opened its own web server category in 2015. In 2022, this competition saw the first teams using deep learning models to predict 3D RNA structures. In the same year, CASP-RNA was launched, a contest co-organized by CASP and RNA-Puzzles (37). It coincided with an explosion of interest in the prediction of the 3D RNA structure (38) resulting, among other things, from the success of AlphaFold and the Covid-19 pandemic caused by an RNA virus. 42 groups participating in CASP-RNA tried their hand at modeling three-dimensional structures for 12 RNA sequences. Eighteen contributing teams used deep learning models (including DeepFoldRNA, RhoFold, trRosettaRNA and OpenComplex-RNA) at various stages of prediction (20–22). The final CASP-RNA ranking gave the top 4 places to teams that combined expert modeling with non-machine learning algorithms.

## THE CHALLENGES

AlphaFold and other highly accurate methods (34,39–48) applied deep learning to predict the protein structure based on the sequence. Training these tools required huge amounts of data. For example, AlphaFold implemented a bootstrap technique in which its final version used both experimentally determined and predicted structures of high accuracy. A fundamental question is whether we have enough RNA structure data for training and whether they are of sufficiently high quality and diversity.

### RNA content in the Protein Data Bank

Since the first tRNA structures were solved in the mid-1970s (49) and published about ten years later (50,51) it was known that RNA molecules could adopt complex 3D architectures. However, it was not until the late 1990s that structures of functionally new types of RNA emerged: first several types of ribozymes (52–54), and then impressive ribosome particles (55–57). These revealed the structural richness of the RNA architectures, which was later confirmed by more structures solved mostly by X-ray crystallography and recently by cryo-electron microscopy (cryo-EM). Despite all the discoveries about RNA structures, the sheer volume of experimental structural data available for RNA and proteins remains strongly in favor of the latter (Table 1). There are about 25 times more protein depositions than RNA. The ratio is slightly more favorable for DNA, but even so, both nucleic acids account for <10% of the PDB archive, and this ratio has remained fairly stable over time. The situation is even more dramatic when restricted to high-resolution data: among X-ray and cryo-EM structures with a resolution better than 2.0 Å, proteins are about 100 times more abundant than RNA (Table 1). Considering all structures with resolution <3.0 Å, RNA nucleotides constitute only 2% of all residues (nucleotides and amino acids) (58,59). Unfortunately, these proportions cannot be expected to change quickly. Newly solved crystal and cryo-em structures tend to have a limited resolution. The reason is the inherent flexibility of RNA molecules that can be estimated, for instance, by factors B and R in the crystal phase; they are higher for RNAs than for proteins with comparable resolution. A limited number of high-resolution RNA structures is a severe constraint, as these structures are the source of the most reliable experimental information about the 3D structures, and some believe the only.

**Table 1.** Numbers of all PDB-released structures (*) and residues in X-ray and cryo-EM structures (**) with high resolution (≤2.0 Å) over decades. In the first column, amino acids are abbreviated as AAs, and nucleotides as nts

	≤1980	1981–1990	1991–2000	2001–2010	2011–2022	Total	% of the total
Proteins (*)	78	634	12 121	43 205	108 677	164 715	91.57
AAs ≤2.0 Å (**)	5050	45 236	1 609 401	11 390 238	28 513 777	41 563 702	99.78
RNA (*)	2	23	306	1392	4488	6211	3.45
RNA nts ≤2.0 Å (**)	0	0	1270	5974	26 921	34 165	0.08
DNA (*)	1	91	1061	2009	5800	8962	4.98
DNA nts ≤2.0 Å	0	238	5430	15 730	38 107	59 505	0.14

### RNA architectures crucial for the global fold

The main architectural element of RNA is an antiparallel double helix of form A that constitutes approximately 60% of RNA in ribosome particles. The structure of this element is the easiest to identify and predict. The overall three-dimensional arrangement of a molecule results from the assembly of these helical regions. It is orchestrated by various types of 3D motifs such as sharp turns, loops, n-way junctions, coaxial stacking of duplexes and triple and quadruple helical regions (56,60). A junction consists of at least three helical regions arranged in a way that significantly influences the overall fold. There are three families of three-way junctions, which differ by the coaxial stacking pattern (60). For junctions with higher multiplicity, it becomes more complicated (61). The correct prediction of the junction topology and the resulting stem orientation is of utmost importance, but poses a significant challenge, as there are usually only single or no homologous junctions in experimental structures of RNA (62). All of the aforementioned regions often form between sequentially distant parts of the RNA molecule and are stabilized by non-Watson–Crick base pairs (NWC). Reliable information on structurally critical NWCs is necessary for the correct 2D/3D structural predictions. However, the collection of NWCs in high-resolution PDB structures is not sufficient to infer their sequence and structural features (63). There are ∼34 thousand RNA nucleotides in high resolution (≤2.0 Å) crystal and cryo-EM structures, compared to ∼42 million amino acids; it is <0.1% of all PDB-deposited residues (Table 1).

**Figure 4.**
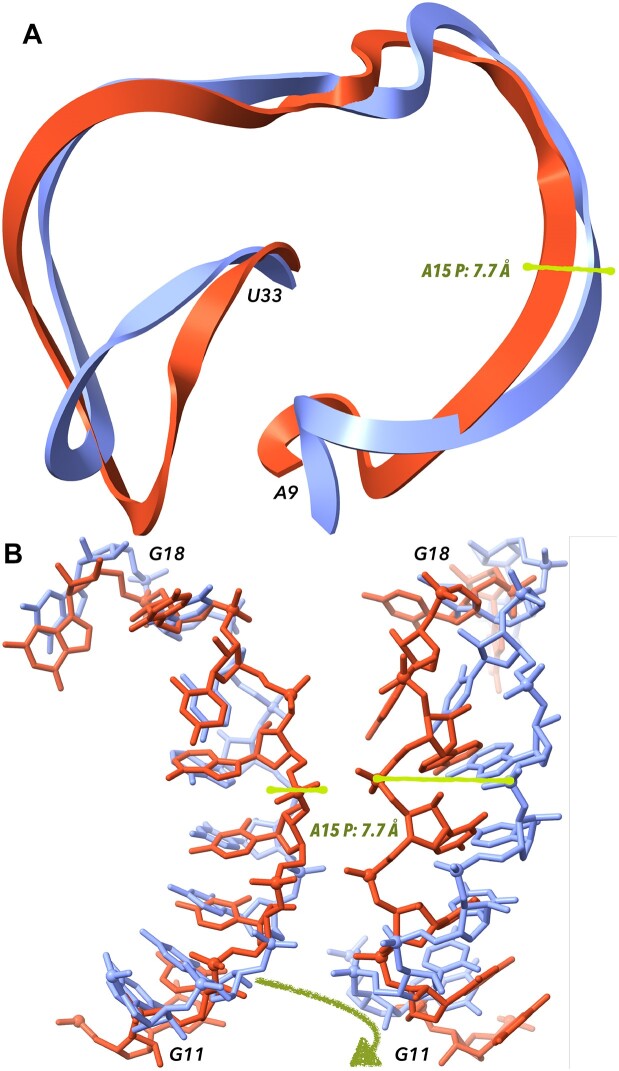
Comparison of predicted and experimentally determined structures. Displayed is hammerhead ribozyme RNA: the structure determined experimentally by X-ray diffraction at the 2.9 Å resolution (PDB ID 5di4) (65) is shown in light blue, the model PZ15_Adamiak_15 is in red. Cartoon representation of the residues A9-U33 in panel (**A**) suggests that the prediction follows the overall topology of the ribozyme correctly but with local deviations. Panel (**B**) shows segments between residues G11 and G18. The overall backbone direction is predicted correctly but local deviations are large. They include differences in base orientations and subsequently in base pairing and also the distances between the corresponding phosphorous atoms are quite large; one such distance between Ps of adenosines 15 of the target and model is highlighted by the green rod. Segments in panel B on the left and right show the same atoms, the view is rotated by ∼90°.

3D modules are another group of crucial yet hard to predict motifs (64) (Figure 4). They are primarily defined by NWCs that form an intricate network of interactions. These networks remain coherent even in RNAs from different phylogenetic groups. 3D modules serve as loops, turns and foundations for protein-RNA or RNA–RNA interactions. Their accurate modeling is essential to catch the global RNA fold, but it is hardly possible due to the low amount of data available.

RNA architectures are also stabilized by interactions such as base-ribose hydrogen bonding, intramolecular interactions with charged phosphates, and coordination with metal ions. The roles of these interactions are even less understood than those of non-Watson–Crick base pairs.

### Quality of experimental RNA data

Not only does the shortage of high-resolution structures complicates the accurate annotation of RNAs. There are problems with the quality of deposited RNA (and DNA) data that arise from the lack of community-accepted quality standards. They are related to base pairing, valence geometry and backbone geometry; their combination can lead to a flood of imprecisely and unreliably refined structures.

A formal description of base pairing is essential to build reliable 3D models. However, base pairing in public archives is not described reliably; it is often incomplete or incorrect. The programs used to assign base pair topology to 3D structures, such as MC-Annotate (66), RNAview (67), FR3D (68), ClaRNA (69), CompAnnotate (69), RNApdbee (70), bpRNA (71), baRNAba (72), BPNET (73) and DSSR (74), often provide incomplete or conflicting information (manuscript in preparation). Therefore, comprehensive benchmarking must be performed along with a consistent update of public archives with topology data from the consensus algorithm(s).

Perhaps of lesser but existing importance for the prediction of large RNA structures is the inconsistency of targets used in the refinement of bond distances and angles. These valence geometry targets differ in various refinement programs, validation packages and the PDB, leading to confusion in the community. Therefore, an ELIXIR-led effort was undertaken by the Nucleic Acid Valence Geometry Working Group (75) to formulate community-agreed validation targets (76–78).

A significant source of errors in the structural description of RNA (and DNA) is the misconception about the geometry of the nucleic acid backbone. The structural complexity of the backbone was understood early on (79), but the topic attracted much less attention until the end of the 1990s. At that time, large RNA ribozyme and ribosome structures started to emerge and it became possible to analyze their structural variability based on experimental data. The smallest unit that makes sense to categorize structurally is a dinucleotide, which includes two riboses and captures the complexity of the phosphodiester linkage C3’–O3’–P–O5’–C5’. However, even this relatively small fragment has nine torsional degrees of freedom. The first conformer definitions of dinucleotide fragments were published at the beginning of 2000, first for RNA (80–82), later for DNA (83) and recently for both RNA and DNA as a structural alphabet CANA built from dinucleotide conformational classes NtC (84). Perhaps the relative novelty of the concept of conformational classes and technical difficulties with their implementation into routine refinement and validation protocols is the reason why the classes are not widely used. We see this fact as one of the reasons why the quality of newly determined structures does not improve.

### Sequences and sequence alignments

The efficiency of 3D RNA structure prediction is likely to be improved using information from multiple sequence alignments (MSA). MSA has already been incorporated into several expert-based modeling methods in the human categories of RNA-Puzzles and CASP-RNA (24). Such a strategy is also applied in AlphaFold and other recent protein prediction methods. In these methods, correlated mutations are used to detect residues that are in close contact in 3D space, despite the distance in sequence. This principle has been understood for a long time in RNA (63). Unfortunately, creating high-quality RNA alignments is difficult and often requires the manual work of an expert. This difficulty has led to there being far fewer RNA vs. protein alignments.

To illustrate the difference in quantity, we can compare two resources, Pfam and Rfam. Pfam and Rfam are collections of protein/RNA alignments and models annotate them in genomes. Rfam is the oldest and largest source of alignments for ncRNAs. Although there are other resources that collect similar data, for example, miRBase (85) or MirGeneDB (86) for RNA, they are smaller and focus on one particular type of molecule. Pfam was founded in 1997 (87), while Rfam in 2003 (88). Each member of Rfam/Pfam is made up of a curated seed alignment which is used to build the model that allows finding more examples of the family and produces what is known as a full alignment. The models in Pfam are based on hidden Markov models, while in Rfam they are covariance models and also include a consensus secondary structure. Here, we will discuss some of the issues facing machine learning practitioners that want to use RNA alignments by comparing these resources.

First, while Rfam is similar to Pfam in spirit and goals, it contains far less data than Pfam. At the time of writing this paper, the current version of Rfam, 14.9, contains 4108 alignments, while the current release of Pfam, 35.0, contains 19 632. The difference in resource size is due to historical bias towards RNA gene discovery, the difficulty in identifying homology between related RNAs, and the difficulty in building new alignments for Rfam. Constructing Rfam alignments requires using covariance models, which are much more computationally expensive compared to the hidden Markov models applied to build Pfam alignments.

**Figure 5.**
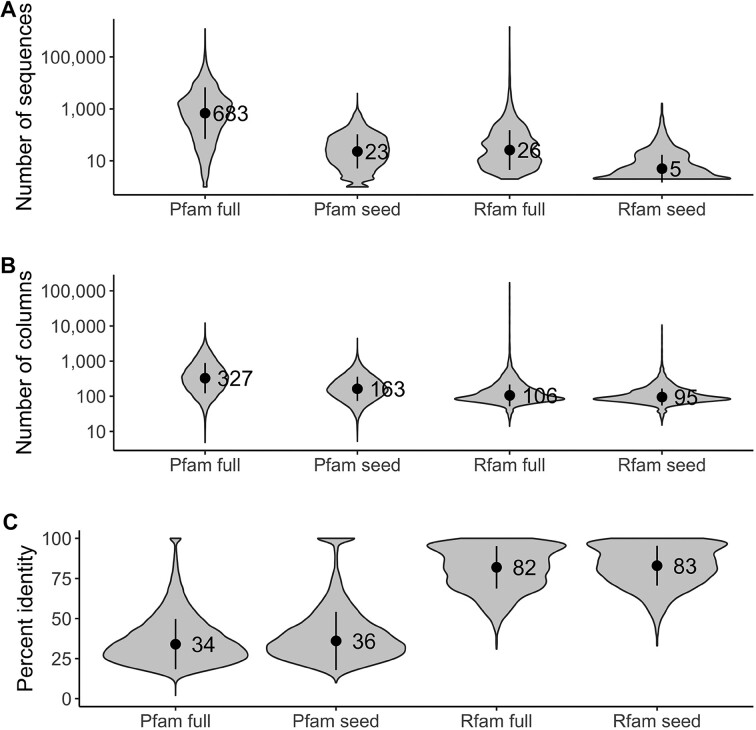
Rfam versus Pfam alignments compared based on (**A**) a number of sequences, (**B**) a number of columns and (**C**) the average pairwise percent identity for each family. The points on the plots indicate the mean, and the vertical bars indicate the standard deviation.

Second, RNA alignments are on average smaller than protein alignments. This relationship relates to the number of sequences, with seed alignments containing an average of 5 sequences in Rfam versus 23 in Pfam (Figure 5A), as well as the number of columns, 95 columns in Rfam versus 163 in Pfam (Figure 5B). There is also a significant difference in the degree of conservation, with the Rfam alignments 83% conserved versus 26% in Pfam (Figure 5C). Together, it means that there are few RNA alignments compared to proteins, and the existing alignments are smaller and lack variation. Therefore, it is likely that there is not enough RNA data yet to effectively train machine learning methods. This is also supported by the fact that the currently best-performing RNA-dedicated methods in CASP are not machine learning based.

**Figure 6.**
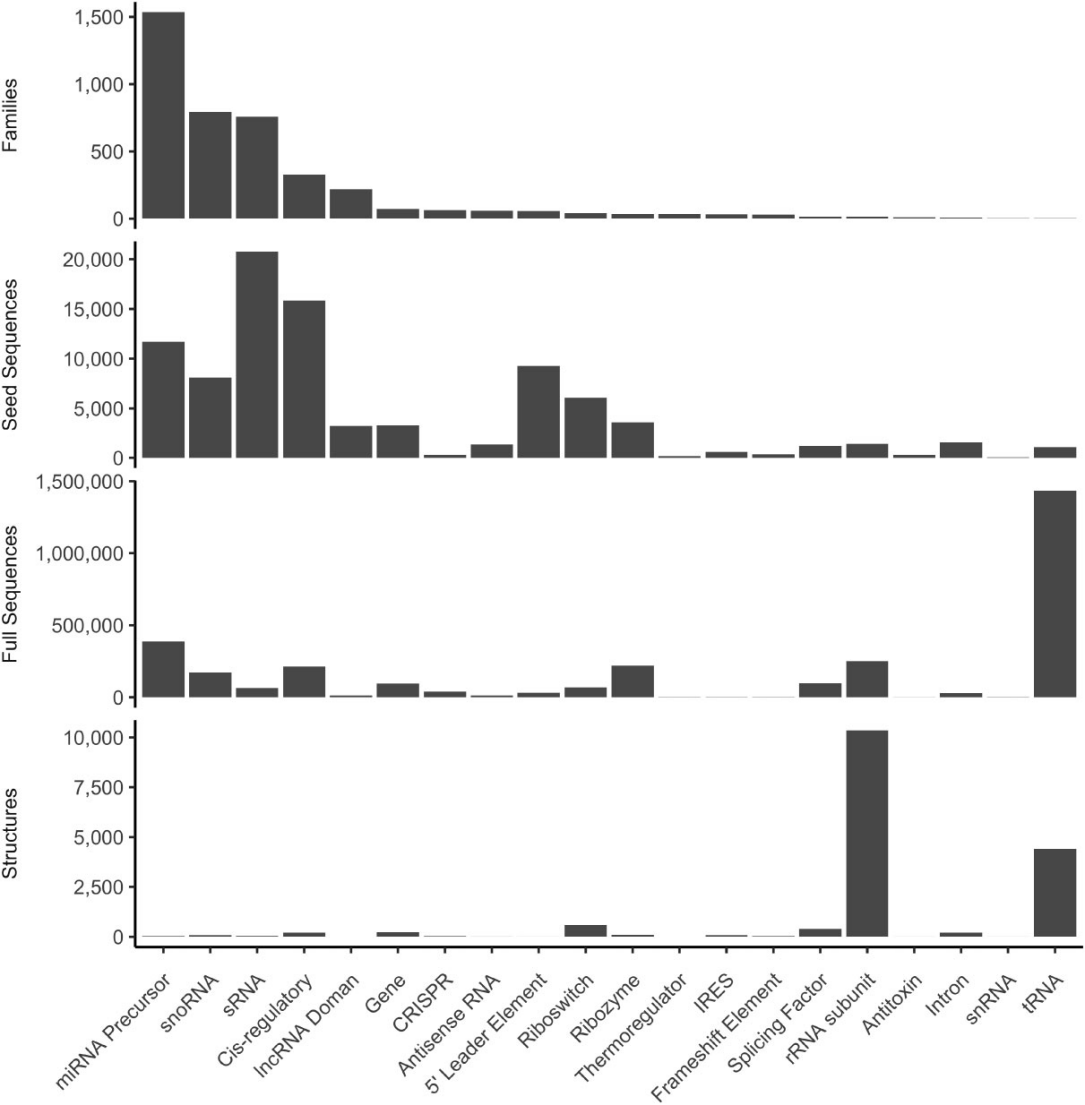
Counts of Rfam families, seed sequences, full sequences and structures for all Rfam families organized by Rfam RNA type.

Third, Rfam alignments have several global biases that make working with them difficult. One is that the most common alignments are for simple molecules. Taking into account the type of RNA, most alignments concern miRNA precursors (35%) followed by snoRNA (19%) (Figure 6). miRNA precursors are simple molecules, essentially a helix with a few small loops and mismatches; in proteins, this is most similar to a single alpha helix. Such simple structures do not represent the complexity of RNA folds; for example, they do not contain any junctions, while—as discussed above—the junction topology is essential to determine the overall structure of more complex RNAs.

Another global bias is observed in the number of seed or full sequences, Rfam has the most data for bacterial small RNA (sRNA) sequences. However, there are few structures of these molecules with <50 in PDB at the time of writing. In terms of full alignments, tRNAs constitute the largest group (45%), and rRNA subunits are the third largest, accounting for another 8% (Figure 6). These families are the most commonly solved structures, representing 26% and 61% of all known 3D structures of RNA, respectively (Figure 6). Although a large collection of these sequences and structures is valuable, we recommend caution. Creating ML models that generalize to other structures is unlikely if their training is based only on ribosomes. Several prediction methods that train off currently existing datasets have not yet produced high-quality models.

In addition to the global bias in the RNA data, there are specific issues with Rfam alignments that must be considered in machine learning. For example, not all non-Watson–Crick base pairs are aligned in Rfam, and the aligned ones have not been handled in a consistent manner. Moreover, Rfam consensus secondary structures can represent parts of the structure as unfolded. However, looking at the 3D structure, when available, in that region often shows a clear secondary structure. These regions include places known to have species-specific structure or their unstructured form results from Rfam limitations. Rfam families are intended to cover a wide phylogenetic range. For example, the eukaryotic large subunit ribosomal RNA family (RF02543) represents all large rRNA subunits in all eukaryotes. However, rRNA is well known to vary considerably within the kingdom, or even within a species, with important functional consequences (89). Since the 2D structures in Rfam must represent what is common to all members of the family, they are often underfolded in many regions. This should be dealt with when building a useful ML training set. Finally, pseudoknots—a key factor in 3D RNA structures—have been shown to help organize the global structure, but are not consistently annotated in Rfam alignments. Unfortunately, current 2D and 3D prediction methods struggle to predict them. Rfam is working to annotate more observed pseudoknots but many families lack them.

In summary, there are several issues with the RNA alignment dataset that will pose problems for deep learning. The data set is small compared to proteins, is highly biased in several ways, and the existing alignments have some shortcomings. While work is ongoing to fix all these issues, it will be challenging to use these data to successfully predict 3D structures. One key issue will be creating a test/train dataset that represents the observed complexity, while not being overly biased.

## CONCLUSIONS

Given the history of protein fold prediction, can we anticipate when the RNA realm will see similar results? AlphaFold’s success came 50 years after the first work on computer-based protein structure prediction. This period of time was necessary to accumulate a sufficient volume of high-quality, reliable data on protein sequences and structures. At the same time, information and computer technology were developed, enabling efficient applications of artificial intelligence models to solve problems that traditional computational methods could not deal with. Artificial neural networks as an idea are already 80 years old (90), but it was only in the second decade of the 21st century that they came into widespread use. In 2012, the power of deep learning was demonstrated (91,92). It has triggered a flood of projects that have applied DL models to various areas of life. Among other things, this wave has brought about new predictive methods dedicated to molecular structures. All of them are data-hungry; AlphaFold has been trained on structures of more than 170,000 proteins combined with very large sequence alignments. We expect to have similar requirements to successfully use neural networks for RNA 3D structure prediction.

A simple way to estimate when AlphaFold for RNA will be created is to consider when the number of RNA structures or sequence alignments are comparable to the currently available protein data. As mentioned above Pfam contains 19 632 protein sequence alignments. Historically, the growth of Rfam has been linear due to the requirement for manual work to build each alignment and we observe that on average Rfam adds approximately 205 alignments per year. Thus, we estimate Rfam will contain 19 000 alignments in approximately 70 years. This is undoubtedly a vast overestimate as we expect the RNA 3D structure prediction problem to be solved by then. One technique which may help is automatic family building. While this is still unsolved for RNA, there has been recent work on this issue which may be promising (93). Automatically built families were used in training AlphaFold and may prove useful for RNA as well (34).

We believe that there are several viable approaches to enable the prediction of the 3D RNA structure in the near future. First, the RNA community can improve knowledge of RNA structure through more data, second, we can diversify the data used in prediction, and finally, we can improve the machine learning methods used.

What data is missing that would improve predictions? We do not seem to know enough about RNA motifs to predict their global structures. We may provide an educated guess, at least for the small structural motifs, of which the most important are base-pair topologies. Concerning the latter, it is very likely that they exist in known structures of reasonably high resolution and can provide reliable geometries. There are also strong reasons to believe that the CANA alphabet describes more than 90% of the existing dinucleotide conformers; only a few of them may be missing (84). In our opinion, more research is needed on intramolecular interactions other than base pairs, namely hydrogen bonding bridges of the O2’ group to bases, ribose, phosphates and interactions between phosphate oxygens (mostly charged) and other RNA constituents. Benchmarking the quality of 3D structures, as well as streamlined and consistent principles of their validation, is required to ensure reliability in data repositories.

Another approach is to improve the size and scope of multiple sequence alignments of RNA. Alignments of four-letter RNA sequences are more challenging than those of 20-letter protein sequences. Some classes of RNA, such as ribosomes, have a large number of sequences and we know how to align them. However, more well-aligned sequences of underrepresented RNA classes are needed. Perhaps the Tree-of-Life projects (94,95) will provide a sufficiently large number of sequences. Currently, RNA gene prediction is inconsistent across known genomes, so we encourage the community to annotate ncRNA genes in newly sequenced genomes. Annotated ncRNAs from Tree of Life projects can show low sequence diversity, and we recommend that ncRNA gene annotation in metagenomes be used as a solution. We note that AlphaFold required metagenomic sequences in order to reach its maximum performance, and we suspect that RNA will show a similar trend. Solving these challenges involves finding all the ncRNA genes and making the data reusable.

Consistently annotating RNA families across all genomes will be useful and may increase the diversity of RNA sequences available; however, it seems that a prediction method would benefit from a wider range of RNA families. As discussed above, many Rfam families are structurally similar. We believe that providing a more diverse training set would be useful. While Rfam is the global repository of RNA families, not all known families can be found there. Correcting this and working to create new families that are different from existing ones should be a focus of the RNA community. Additionally, creating high-quality alignments remains a challenge (96).

If the current amount and growth rate of currently available sequence and structure data are not sufficient, can they be supplemented with other sources of data? We think so. In particular, RNA biochemistry has a rich history and has developed many methods to rapidly probe 3D structures (97,98). A subset of these data, SHAPE probing, has proven useful to classical prediction methods, and we expect it to be helpful to DL-based approaches. Although many labs probe the structure of RNA, these data are not readily available to ML practitioners. Working as a community to standardize, collect and distribute such data seems valuable for predictions. Additionally, there are other low resolution methods, such as SAXS and AFM, which may prove useful in modelling structures (97).

Finally, the rapid and hard-to-predict development of ML methods may potentially change our pessimistic predictions about the ability to accurately predict 3D RNA structures. Development of methods that are less data hungry, e.g. transfer learning, may allow successful prediction sooner. We believe that RNA structure prediction is an excellent test case for researchers interested in machine learning in the face of limited data. At the moment, we do not believe that reliable 3D RNA prediction will be available in the 2020s, but we challenge the community to prove us wrong.

## Data Availability

The data underlying this article are available in Zenodo, at https://doi.org/10.5281/zenodo.8167407.
